# Factors Associated with the Level of Physical Activity in Middle-Aged Colombian People during Lockdown in Response to COVID-19: A Cross-Sectional Study

**DOI:** 10.3390/healthcare10061050

**Published:** 2022-06-05

**Authors:** Patricia Alexandra García-Garro, Agustín Aibar-Almazán, Yulieth Rivas-Campo, Gloria Cecilia Vega-Ávila, Diego Fernando Afanador-Restrepo, Antonio Martínez-Amat, María Isabel Afanador-Rodríguez, Fidel Hita-Contreras

**Affiliations:** 1 GIP Pedagogy Research Group, Faculty of Distance and Virtual Education, Antonio José Camacho University Institution, Santiago de Cali 760016, Colombia; palexandragarcia@admon.uniajc.edu.co (P.A.G.-G.); yrivasc@usbcali.edu.co (Y.R.-C.); gcvega@profesores.uniajc.edu.co (G.C.V.-Á.); dafanador4@areandina.edu.co (D.F.A.-R.); maria.isabel.afanador@gmail.com (M.I.A.-R.); 2 Department of Health Sciences, Faculty of Health Sciences, University of Jaén, 23071 Jaén, Spain; amamat@ujaen.es (A.M.-A.); fhita@ujaen.es (F.H.-C.)

**Keywords:** physical activity, sleep quality, depressive symptoms, quality of life, lockdown, COVID-19

## Abstract

(1) Background: Due to the pandemic caused by COVID-19, mandatory confinement was declared, which generated a decrease in the practice of physical activity (PA). Based on this problem, it was proposed to study the associations between PA in relation to depressive symptoms, quality of sleep, and the quality of life of middle-aged people who work in the university context during compulsory confinement as a result of COVID-19. (2) Methods: A total of 336 middle-aged people (48 ± 6.67) participated in this analytical cross-sectional study. The variable levels of PA, quality of sleep, symptoms of depression, and quality of life were measured with the International Physical Activity Questionary (IPAQ), the Pittsburgh Sleep Quality Index (PSQI), the Zung Self-Rating Depression Scale (ZSDS), and the SF-12v2 questionnaire, respectively. (3) Results: A logistic regression model was used to analyze the relationships between the level of PA and depressive symptoms (OR = 2.053), total sleep duration (OR = 0.495), sleep disturbances (OR = 2.414), quality of sleep (OR = 2.471), use of sleep medication (OR = 0.348), daytime dysfunction (OR = 1.809), general health (OR = 0.949), and physical functioning (OR = 0.987). (4) Conclusions: In middle-aged people, during compulsory confinement, being insufficiently active is a risk for depressive symptoms and disturbances in sleep quality.

## 1. Introduction

In December 2019, a new highly contagious, respiratory, infectious disease emerged in Wuhan, China, caused by SARS coronavirus 2 (SARS-CoV-2) [1], a disease now known as COVID-19. This virus spread rapidly throughout the world, causing significant morbidity and mortality, and unleashed a global health emergency. On 11 March 2020, the World Health Organization (WHO) declared COVID-19 a pandemic [2]. The scale of the pandemic has generated great concern around the world due to its social and economic impacts [3] and its impacts on the Health-Related Quality of Life (HRQoL) of people. HRQoL has become an important goal to be achieved in collective health and encompasses the subjective and multidimensional concepts of physical, mental, and social health [4]. Thus, when responding to the health crisis caused by COVID-19, the health of the general population should be considered.

Many countries, including Colombia, adopted measures to protect people, through home confinement and social distancing, and thus mitigated the speed of spread of the virus [5]. In this way, limiting interpersonal relationships has generated nonconformity, rejection, and even aggressiveness towards the measures adopted. In addition, due to the reprocessing of the basic systems of population functioning, loss of lives, instability in the health system, dissatisfaction in the provision of services, economic stagnation, inequity, deficient government support, and scarcity of options for economic reactivation, episodes of depression and decrease in the HRQoL of the Colombian population have been evidenced [6]. On the other hand, major changes were generated in the activities of daily living. For example, the time dedicated to performing PA was reduced due to restrictions on participation in outdoor activities, and travel to schools, universities, and workplaces [7]. These measures have led to multiple negative effects on HRQoL [8]. An investigation with a Chinese population reported that the most frequent problems related to HRQoL during the lockdown were pain/discomfort (19.0%) and anxiety/depression (17.6%) [9]. On the other hand, studies carried out during previous epidemics have registered that a third of the population that experiences social isolation develops insomnia. During the lockdowns, sleep pattern alterations increased, with a prevalence of 33.7%; such alterations can lead to mental disturbances, such as anxiety disorders, depression, and post-traumatic stress disorder [10].

In the same way, the need for physical isolation generated a change in work dynamics, and the personnel of Colombian universities were no exception; they went from operating face to face to remotely. This practice brings with it some disadvantages, such as social isolation, presenteeism, the perception of lack of support (in relation to technical support), and blurring the limits of the workload [11]. These disadvantages appeared to be exacerbated in the context of the lockdown, due to the confinement and stress generated by this situation. It has been suggested that the home work modality could generate physical and mental health problems in Colombian professionals [12], thereby affecting HRQoL. However, few scientific studies have addressed this issue in depth. For this reason, in order to preserve HRQoL, while taking into account the multiple benefits that PA has on physical and mental health, which has been widely documented [13], physical training at home was recommended during the coronavirus outbreak. In this way, some studies showed a general increase in PA in Belgium and Canada. However, an overall decrease in PA was observed in people from Asia, America, Africa, and Europe [14].

Preliminary evidence during the COVID-19 outbreak suggests positive associations between PA and physical and mental health, and inverse associations between sedentary behavior and physical and mental health outcomes [3]. A study conducted in Japan found that lower HRQoL scores for the mental component were associated with an increased risk of decreased PA in older adults [15]. Additionally, a study carried out in Australian adults concluded that changes in PA, sleep, smoking, and alcohol intake are associated with greater depression, symptoms of anxiety, and stress [3]. For its part, a study carried out with Ukrainian university students found that the inactive group had higher anxiety and depression scores than the physically active group during the pandemic. The relationship of PA with anxiety and depression was statistically significant but weak [14]. Currently, there is no available research on the associations of PA and HRQoL in middle-aged university personnel, during compulsory confinement due to COVID-19. This type of research may be used to screen risks associated with health and to generate prevention and promotion programs for health and well-being for this population.

Therefore, the objective of this study was to examine the associations between PA, HRQoL, depression symptoms, and sleep quality in middle-aged Colombian University personnel, during home confinement, as a result of the COVID-19 health crisis. Likewise, being physically active is expected to be associated with better HRQoL and sleep quality as well as the absence of depressive symptoms; similarly, being insufficiently active is expected to be associated with worse HRQoL and sleep quality as well as the presence of depressive symptoms in middle-aged Colombian university personnel during mandatory confinement due to COVID-19.

## 2. Materials and Methods

### 2.1. Study Design and Participants

An analytical cross-sectional study was carried out involving university personnel in Cali, Colombia. The mandatory lockdown was implemented in Colombia from 25 March 2020 to 31 August 2020. The data collection was conducted between 20 May and 10 June of the same year. The mandatory confinement limited the free movement of people and vehicles in the national territory with 34 exceptions that seek to guarantee the right to life, health, and survival of the country’s inhabitants as part of the instructions issued due to the health emergency generated by the COVID-19 pandemic; however, university personnel were not included in these exceptions. Due to this limitation of mobility, all the educational and administrative activities of the multiple institutions were carried out remotely.

**Figure 1:**
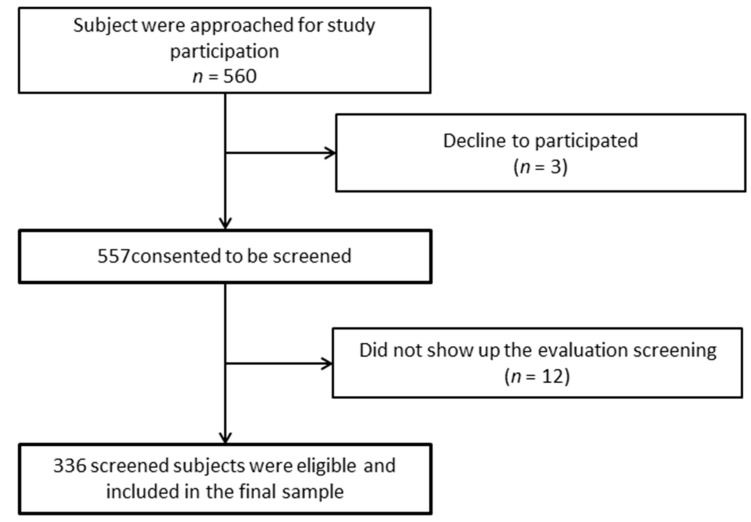
Flow diagram of study design.

A total of 336 people participated in this study (Figure 1). The inclusion criteria dictated that participants had to be people between 40 and 64 years old; had to be teaching staff or administration and services staff of a university; and had to sign informed consent. Prior to study enrollment, all participants received a video with detailed study information and gave their written consent through an online form. This study was approved by the Research Ethics Committee of the Antonio José Camacho University Institution (FEDV-001-21-01).

### 2.2. Outcomes

All the variables in this study were measured through an online form due to the current situation in the country.

#### 2.2.1. Level of Physical Activity

The short version of the International Physical Activity Questionnaire (IPAQ) is a frequently used instrument to measure PA for 7 days prior to assessment and is reported to have high reliability. The Colombian version of the IPAQ was translated and validated in the Colombian population by Craig et al. [16] (criterion validity had a median rho of about 0.30). This questionnaire provides information about the time that the person spends in doing activities of moderate and vigorous intensity, in walking and in sitting; and assesses three characteristics of PA: intensity (mild, moderate or vigorous), frequency (days per week), and duration (time per day). Weekly activity is recorded in Mets (metabolic equivalents of a task) per minute and week, and its reference values are: walking—3.3 Mets; moderate PA: 4 Mets; and vigorous PA: 8 Mets. To obtain the number of Mets, each of the previously mentioned values (3.3, 4 or 8 Mets) must be multiplied by the time in minutes of the activity being carried out in a day and by the number of days a week it is carried out [17]. The results were summarized in three categories: high, which is defined as 3 days or more of vigorous activity reaching an energy expenditure of 1500 Mets × min/week or as 7 days or more of moderate activity with an energy expenditure of at least 3000 Mets × min/week; moderate, defined as 5 days or more of moderate activity with an energy expenditure of at least 600 Mets × min/week, 5 days or more of moderate activity at least 30 min/day or 3 days or more of vigorous PA for at least 25 min/day; and finally, low, which is defined as when the other two categories are not met or when no activity is performed.

The results obtained from the IPAQ classification were recategorized as a binary outcome: insufficiently active or active. Those who were classified as having high and moderate levels of PA were considered active; those who obtained a low level were recategorized as insufficiently active.

#### 2.2.2. Quality of Life Related to Health

The SF-12v2 [18] questionnaire measures health-related quality of life, which has been validated for the Colombian population (Cronbach’s Alpha = 0.7) [4]. This questionnaire is composed of 12 items that define a positive or negative state of physical and mental health through eight domains or scales: (i) physical functioning, (ii) physical role, (iii) bodily pain, (iv) general health, (v) vitality, (vi) social functioning, (vii) emotional role, and (viii) mental health. In addition, it generates two other summary scores: a physical component summary and a mental component summary. The total score ranges between 0 and 100, and a higher score implies a better health-related quality of life.

#### 2.2.3. Sleep Quality

The sleep quality was assessed with the Pittsburgh Sleep Quality Index (PSQI) [19] validated for the Colombian population (Cronbach’s Alpha = 0.78) [20], composed of a total of 24 questions of which 19 are self-evaluated questions and 5 must be answered by a bed or roommate. This questionnaire generates a total score and 7 domains or subscales: (i) subjective sleep quality, (ii) sleep latency, (iii) sleep duration, (iv) habitual sleep efficiency, (v) sleep disturbances, (vi) use of sleep medications, and (vii) dysfunctions during the day. These questions each have a scale of 0 to 3, with higher scores indicating poorer sleep quality. In turn, the total score ranges from 0 to 21, with >5 being a poor quality of sleep [19,20,21].

#### 2.2.4. Depression

Depression was evaluated by the Zung Self-Rating Depression Scale (ZSDS) (Cronbach’s Alpha = 0.85) [22], which consists of a self-administered survey that identifies the level of depression in adults and has been validated for the Colombian population by Campo-Arias et al. [23]. This scale consists of a total of 20 questions, of which 10 are positive and the other 10 are negative. Each question is evaluated on a scale of 1–4 and refers to the frequency of depressive symptoms in the last 2 weeks, indicating the four characteristics of depression: the dominant effect, the physiological equivalents, other disturbances, and psychomotor activities. Their total score ranges from 20 to 80 points, where a higher score implies worse depression. Anyone who scored 50 or more points on the ZSDS was categorized as having depressive symptoms, those below this score were perceived as having no depressive symptoms.

### 2.3. Covariates

All the covariables in this study were measured through an online form due to the current situation in the country.

#### Sociodemographic and Anthropometric Data

All study participants answered a series of questions about demographic data, such as age, sex, marital status, labor relations, socioeconomic strata, housemates and geographical location of residence (According to DANE [24]) or urban area of residence, risk patient, smoking habits and alcohol, height, and weight. The body mass index (BMI) was obtained by dividing the weight of a participant (kg) by their height squared (m^2^).

### 2.4. Sample Size Calculation

The sample size was obtained with the following parameters: from a total population of 416 people, with 95% reliability, 3% precision, and 50% expected prevalence (insufficiently active), under a random sampling design based on the Epidat 4.1 software (Dirección Xeral de Innovación e Xestión da Saúde Pública de la Consellería de Sanidade with the support of the Pan American Health Organization, Coruña, Spain) with a design effect adjustment of 1%, 300 people were the result; however, it was adjusted to a non-response percentage of 10% for a total of 330 people required.

Likewise, the calculation of 10 participants per variable was supported for the logistic regression model. Given that we used 19 possible predictor variables related to sleep quality (7 domains and the total score of the PSQI), quality of life (11 categories), and depression (ZSDS), in addition to 11 sociodemographic covariates, more than 300 subjects were required for the purposes of our analysis. The final number of participants was 336.

### 2.5. Data Analysis

Exploratory data analysis was performed to identify missing values. The questionnaires used for data collection were completely filled out; therefore, significatively there was no missing data (Figure 1). The distribution of the independent variables and outcome were described, in relation to the characteristics of randomness and normal distribution (from the Kolmogorov–Smirnov test), and the identification of extreme values.

From the univariate analysis, we proceeded to characterize the study population; mean (m) and standard deviation (SD) were used for continuous variables due to their distributions being adjusted to normal. The qualitative variables of the sociodemographic area, the level of PA, the level of depression, and the total scores of the SF-12v2 and PSQI were presented through their frequency (*n*) and percentage (%) in each category. This allowed us to present a descriptive analysis of the result variables and each of the independent variables. The IPAQ variable was recategorized into two options: active and insufficiently active. From them, bivariate analysis was carried out, where the significant differences between the levels of PA coinciding with each of the variables and covariates were identified. For the analysis of quantitative variables by their distribution and equality of variances, the *t*-test was used, identifying whether or not there were differences between the means of the groups (active and insufficiently active). The categorical variables were tabulated according to the exposure group and analyzed using a Chi2 or Fisher test. For all statistical tests will a null hypothesis, a significance level of 0.05 and a confidence level of 95% (95% CI) were established. A simple regression model was established for each independent variable, allowing one to know the individual association measure (OR) with the IPAQ level.

In multiple analyses, confounding factors for age, sex, and occupation were ruled out. Differences were evaluated between each crude OR and each one adjusted by the Mantel–Hanzel test. For the results found in bivariate analysis, those that presented significant differences (*p* < 0.05) were integrated into a logistic regression model. The chance ratio (OR) was considered significant when it was different from 1 and when the 95% CI did not include 1. Considering the principle of parsimony, the goodness of fit was evaluated by looking for the model with fewer variables. For them, the model comparison test (likelihood ratio test) with the backward method was used. Finally, the post-estimation was carried out with the Hosmer-Lemeshow test.

## 3. Results

The descriptive data of the population are presented in Table 1. In total, the sample was made up of 336 participants (55.65% men), who had a mean age of 48.8 years (±6.67) and a mean of 26.5 (±4.1) for BMI. Aside from BMI, 79.46% did not present other comorbidity risks for COVID-19, 44.9% were married, 69.94% were teachers, 97.62% lived in urban areas, and 50.3% belonged to the low socioeconomic stratum. The study population presented a mean of 86 (±14.2) in the total HRQoL score and a mean of 7.02 (±3.7) in the total PSQI score; 75.6% were found to not have symptoms of depression and 54.76% were classified as insufficiently active.

Table 2 shows mean differences between people classified as active and insufficiently active, showing significant differences in HRQoL related to the following domains of the SF12v2: physical functioning (*p* < 0.001), physical role (*p* < 0.001), bodily pain (*p* = 0.009), general health (*p* < 0.001), physical health component summary (PCS; *p* < 0.001), vitality (*p* = 0.012), emotional role (*p* < 0.001), and total score (*p* = 0.002). Regarding the PSQI, differences were found in all its domains (*p* < 0.05), which shows the similarity of its variables with those in the IPAQ.

Regarding the independent associations with the IPAQ, the simple logistic regression model showed that being insufficiently active during the confinement period was associated with presenting depressive symptoms (OR = 3.35, CI = 1.9–5.8); being insufficiently active was 3.35 times more common in those who presented symptoms of depression than in those who did not. For the SF-12v2 categories, a protective association was found: general health (OR = 0.94, CI = 0.92–0.95); physical functioning (OR 0.97, CI = 0.95–0.98); physical role (OR 0.98, CI = 0.97–0.99); emotional role (OR 0.98, CI = 0.97–0.98); bodily pain (OR 0.98, CI = 0.96–0.99); vitality (OR 0.98, CI = 0.97–0.98); and physical summary (OR 0.98, CI = 0.98–0.988) show that the higher the scores in these HRQoL domains, the less the chance of being insufficiently active. For its part, for the results of the PSQI, direct associations were found between its total score (OR 1.31, CI = 1.2–1.4) and each of its components (*p* < 0.05), showing that the greater the difficulty of sleep, the greater the chance of being insufficiently active (Table 3). These variables were evaluated with the Mantel and Hanzel test to rule out confusion and modification of the effect, showing convergence of the crude and adjusted OR even when stratified by categories of age groups and sex; they were then passed to the analysis of the multivariate model.

The multiple logistic regression model evaluated all the variables that were significantly related in the bivariate model. Subsequently and while continuing with the search for the most parsimonious model, the backward method was carried out, wherein a model is summarized with the most significant variables that explain the association. The final multiple logistic regression model (Table 4) indicated that during the period of confinement due to the COVID-19 pandemic, the IPAQ was associated with the dimensions of the SF-12v2: general health (OR = 0.94, CI = 0.92–0.97) and physical functioning (OR = 0.98, CI = 0.97–0.98). It was also associated with the result of the ZSDS (OR = 2.05, CI = 1.9–4.5). Regarding the results of the PSQI, an association was found with the components sleep efficiency (OR = 2.41, CI = 1.37–4.22), sleep disturbances (OR = 2.47, CI = 1.28–4.75), use of sleeping medication (OR = 4.34, CI = 1.99–9.49), and daytime dysfunction domain (OR = 1.80, CI = 1.23–3.19). The goodness of fit test was performed, with the Hosmer–Lemeshow test revealing that this model is appropriate (Chi^2^ = 1.32, *p* = 0.607) and that the model was classified correctly (80.65%).

## 4. Discussion

This work provides information on how the level of PA was associated with different variables related to the well-being of people working in the Colombian university context, during the period of mandatory confinement as a result of the COVID-19 health crisis. For this, sociodemographic variables, HRQoL, sleep quality, and symptoms of depression were taken into account. This information was gathered due to the need to generate strategies to promote healthy lifestyles and to monitor the health and well-being of this population in times of pandemic.

Numerous investigations have reported significant changes in both lifestyles [25] and social behavior [26] during the COVID-19 pandemic, with a significant increase in the mean sedentary time [27] and a reduction in physical activity both being evident in different populations [28,29]. In contrast, Cabrera, E. (2020) [30] proposed that during the pandemic, physical activity has become a media phenomenon, which has allowed the proliferation of exercise programs of all kinds and in different platforms and media, with the aim of mitigating the emotional imbalances of confinement and favoring the maintenance of health. Thus, some studies have shown an increase in BP levels during the pandemic [31,32].

In the case of workers, it was reported that in Italy the participants (44.9 ± 13.3 years) reduced their frequency of PA before and after the pandemic from 51.2% to 32.2%, respectively [33]. This is consistent with our findings, since during confinement more than half (54.76%) of the respondents did not meet the minimum recommendations of PA, instead being classified as insufficiently active. It is worth noting that public health measures have affected differently people who were active and inactive before the pandemic, generating a greater impact on those who were active [32,34]. Furthermore, we found that being a woman and being married increased the opportunity of being insufficiently active by 1.4 and 1.7 times, respectively, which coincides with previous reports that showed higher levels of PA in men during the pandemic [35], and that the levels of PA were higher among unmarried people of both sexes [36]. On the other hand, according to the reference standards for the Colombian population [37], we found that those evaluated were overweight; however, there were no significant differences in the BMI between those classified as insufficiently active and the active ones. These results are in line with a Brazilian report; the BMI did not distinguish between those who performed vigorous PA and those who did not, during the COVID-19 pandemic [38].

Using the SF-12 questionnaire, we found that the general population maintained a good quality of life; this is in line with studies carried out on the Chinese adult population, where positive effects on quality of life and mental health were reported during the lockdowns [9,38]. One reason for these findings could be that during this type of situation, people pay greater attention to their mental and physical health [39]; it is also possible that they develop resilience mechanisms to face the stress generated by the crisis, improving their perception of well-being and health [40,41]. Likewise, when inquiring into the scientific literature, we must note that HRQoL during the pandemic worsened in people with chronic health problems [42], and in those who have had this disease [43], compared to those who never developed it, and in people who they were unemployed during confinement [44]. It should be noted that, in our sample, no person with COVID-19 was found; only 20.5% reported having one or more relevant medical conditions; in addition, all those evaluated had their employment assured, and this could explain the results obtained in relation to the perception of health.

Our results showed an association between HRQoL and PA, especially related to the domains: physical functioning, physical role, emotional role, body pain and vitality, and with the physical summary. Consistent with our results, a study conducted with Canadian adults showed that physical activity was strongly associated with emotional, psychological, and social well-being [32]. Similarly, Zuzuki et al. [15] reported that low scores in the mental summary component subdomain were associated with a decrease in PA in older Japanese adults.

Additionally, during confinement, we found that the population had difficulty in sleeping, also PA levels and sleep problems were related, so the greater the difficulty in sleeping, the greater the likelihood of being insufficiently active. Consistently with our findings, Targa et al. [45] reported that 71 Spaniards (40.7 ± 11.9 years) increased their score on the PSQI scale by 13.4% during the COVID-19 lockdown, thereby demonstrating a worsening of the quality of sleep. It has also been reported that changes induced by the pandemic exacerbated sleep problems, leading to an increase in reports of people with distress of this type, worldwide [46]. There were high prevalences of insomnia and daytime sleepiness in some populations [47]. This situation could be partially explained by modifications in wakefulness and sleep habits that are potentially harmful to health, to the point that in 2020 a group of experts suggested strategies to improve sleep quality in the technical guidance for the general public regarding COVID-19 measures [48]. In addition, it is believed that the deterioration in the quality of sleep affects women more negatively. It is associated with various factors, such as a reduction in PA, being between 31 and 45 years old, being in home confinement, and presenting a reduction in economic situation [49]. Although the dose–response relationship between the amount of PA and good quality of sleep has not been clarified [50], it has been reported that higher levels of PA for prolonged periods of time are shown as a protective factor against the development of insomnia in women; in previously sedentary men, an 8-week program of moderate–high-intensity aerobic exercise increased nocturnal salivary melatonin levels while improving their total PSQI score [51].

On the other hand, we found 24.4% prevalence of depressive symptoms, which was similar to that reported in American adults (26.5%) [52]; the prevalence appeared to increase in people aged 35–49 years old and in people with poor quality sleep and lack of PA [53]. A study with 1723 workers from Arab higher institutions (37.4 ± 13.4 years) revealed that factors such as PA, diet, and sleep patterns were associated with mental well-being during COVID-19 confinement [54]. Consistently with those results, we found that for participants with depressive symptoms, the chance of being insufficiently active was significantly increased. According to Ramírez-Ortiz et al. [55], the epidemic increased psychological disorders in the general population, meaning epidemic outbreaks could lead to deterioration in mental health.

In addition, it is important to note that there is a bidirectional reciprocal relationship between PA and sleep quality [56,57], as well as between PA and depression symptoms [58,59]; therefore, they may influence each other in such a way that poor sleep quality may contribute to low PA levels [60] and the presence of depression symptoms may contribute to low PA levels [59]; this bidirectional relationship has been observed in contexts other than the COVID-19 lockdown [61,62].

Physical activity has been shown to improve physical and mental health and well-being [63]. Recent research found that increased aerobic capacity could prevent COVID-19 or lessen its severity [64]. Thus, people with higher levels of PA have better HRQoL [43], better quality of sleep [25], and fewer symptoms of depression [51,52,53]. Despite the fact that there are some previous studies that link PA with either HRQoL, or with the quality of sleep and/or the reduction of depressive symptoms during the pandemic, to our knowledge, there have been no studies carried out in the middle-aged Colombian population who work in the university context, during the mandatory confinement caused by this pandemic. We emphasize that most of the investigations that have been carried out in the Colombian population, in this context, have examined aspects related to the biology of the virus, the epidemiology of the infection, the mortality of the infection, suicide risk, depression, perceived stress, resilience to disease and the impacts of infection on children and adolescents, and those who work in hospitals [65,66,67]. Therefore, there is clearly a gap in knowledge in relation to the population of interest that we are addressing. The number of factors examined in this work allows a more comprehensive analysis of the situation of this population and, therefore, constitutes another strength of our study.

Finally, some limitations were identified. Previously studied variables related with the pandemic, such as anxiety levels, were not examined. However, the Hosmer–Lemeshow goodness-of-fit test revealed the suitability of this model and its correct classification. Additionally, since this was a cross-sectional study, causal relationships could not be made. Similarly, no data were collected prior to mandatory confinement, so it is not possible to make a comparison prior to confinement and during confinement. Although the sample was large and representative, it only generated data from questionnaires; therefore, the influence of recall bias cannot be ruled out.

## 5. Conclusions

This study confirmed that a high percentage of middle-aged university staff had a low level of PA during mandatory confinement due to COVID-19. It was shown that the higher the HRQoL, the lower the chance of being insufficiently active; on the other hand, the deterioration in sleep quality and the appearance of depressive symptoms were identified as factors associated with presenting a low level of PA.

The results of this study suggest that PA is potentially useful for facing the adverse effects generated by the lockdowns on HRQoL, sleep quality, and the increase in symptoms of depression; in addition, it can be used to generate programs of prevention and health promotion that promote the well-being of this population.

## Figures and Tables

**Table 1:** Descriptive data of the sample.

Study Sample (*n* = 336)			
Age. mean (SD)			48.80 (6.67)
Sex. *n* (%)	Male	187 (55.65)	
	Female	149 (44.35)	
BMI. mean (SD)			26.50 (4.11)
* Risk patient. *n* (%)	No		267 (79.46)
	Yes		69 (20.54)
Housemates. mean (SD)			1.28 (1.18)
Marital status. *n* (%)	Single/Free Union		132 (39.29)
	Married		151 (44.94)
	Separated /widower		53 (15.77)
Labor relations. *n* (%)	Teacher		235 (69.94)
	Administrative		101 (30.06)
Residence. *n* (%)	Rural area		8 (2.38)
	Urban area		328 (97.62)
** Socioeconomic strata. *n* (%)	Low		169 (50.30)
	Medium		107 (31.85)
	High		60 (17.86)
Smoker. *n* (%)	No		326 (97.02)
	Yes		10 (2.98)
Alcohol. *n* (%)	Not consumption		143 (42.56)
	Yes consumption		193 (57.44)
	Frequency	Occasional	189 (56.25)
		Frequently	4 (1.19)
SF-12v2. mean (SD)	Physical Health dimension	Physical functioning	86.97 (21.67)
		Physical role	82.73 (33.67)
		Bodily pain	91.22 (17.19)
		General health	75.00 (15.81)
		PCS	344.63 (64.79)
	Mental Health dimension	Vitality	81.60 (19.14)
		Social functioning	89.65 (19.86)
		Emotional role	85.11 (32.58)
		Mental health	79.59 (18.12)
		MCS	341.97 (59.41)
	Total score for SF-12v2		86.02 (14.20)
PSQI. mean (SD)	Sleep quality domain		1.12 (0.72)
	Sleep latency domain		1.38 (0.99)
	Sleep duration domain		1.37 (0.81)
	Sleep efficiency domain		0.51 (0.68)
	Sleep disturbances domain		1.32 (0.62)
	Use of sleeping medication domain		0.46 (0.64)
	Daytime dysfunction domain		0.86 (0.72)
	Total score for PSQI		7.02 (3.78)
ZSDS. *n* (%)	Without (DS)		254 (75.60)
	With (DS)		82 (24.40)
IPAQ. *n* (%)	Active		152 (45.24)
		High	84 (25.00)
		Moderate	68 (20.24)
	Insufficiently active	Low	184 (54.76)

BMI (kg/m^2^): body mass index. SF-12v2: 12-item short-form health survey. PCS: physical health component summary. MCS: mental health component summary. PSQI: Pittsburgh Sleep Quality index. ZSDS: Zung Self-Rating Depression Scale. DS: depressive symptoms. IPAQ: The International Physical Activity Questionnaire. SD: standard deviation. * Have one or more underlying medical condition for COVID-19, such as asthma, diabetes, high blood pressure, cancer, a chronic kidney disease, rheumatism, cardiovascular disease, hematological disease, or orphan disease. ** According to Law 142 of 1994 that establishes the Regime of Domiciliary Public Services in Colombia [24].

**Table 2:** Differences of means according to the level of physical activity.

Independent Variable			OR	P > z	[95% CI]		OR Age Adjusted	P > z	[95% CI]		OR Sex Adjusted	P > z	[95% CI]	
					Min Max				Min Max				Min Max	
Sociodemographic	Age		0.99	0.739	0.96	1.02	-	-	-	-	0.98	0.746	0.97	1.02
	Sex male		0.73	0.158	0.47	1.12	0.72	0.178	0.47	1.12	-	-	-	-
	+Sex Female		1.44	0.028	1.04	1.99	1.42	0.029	1.04	2.00	1.44	0.031	1.09	1.89
	BMI		1.01	0.645	0.96	1.06	1.06	0.648	0.96	1.06	1.06	0.645	0.96	1.06
	Residence rural area		0.82	0.784	0.20	3.34	0.83	0.790	0.20	3.34	0.84	0.784	0.20	3.34
	+Urban area		1.21	0.078	0.97	1.51	1.23	0.01	0.97	1.51	1.29	0.089	0.97	1.71
	Stratification													
		High	1.25	0.456	0.68	2.28	1.25	0.466	0.68	2.28	1.23	0.456	0.68	2.28
		Medium	0.91	0.733	0.56	1.49	0.93	0.753	0.56	1.49	0.93	0.733	0.56	1.49
		+Low	1.19	0.249	0.88	1.61	1.20	0.250	0.88	1.61	1.17	0.257	0.98	1.58
	Marital status													
		Married	1.76	0.018	1.10	2.83	1.77	0.018	1.10	2.83	1.78	0.028	1.10	2.86
		Separated/widower	1.77	0.083	0.92	3.39	1.77	0.080	0.92	3.90	1.76	0.073	0.99	3.40
		+Single/Free Union	0.85	0.385	0.61	1.20	0.86	0.389	0.61	1.20	0.85	0.450	0.61	1.40
	Smoker		0.08	0.021	0.01	0.69	0.07	0.026	0.01	0.69	0.09	0.021	0.01	0.69
	+No Smoker		1.27	0.027	1.02	1.59	1.25	0.029	1.02	1.59	1.25	0.027	1.02	1.59
	With * risk		1.36	0.254	0.79	2.34	1.38	0.255	0.79	2.34	1.37	0.254	0.79	2.34
	+Without * risk		1.13	0.298	0.89	1.44	1.15	0.299	0.89	1.44	1.10	0.298	0.89	1.44
	Alcohol		0.92	0.708	0.59	1.42	0.94	0.710	0.59	1.42	0.91	0.708	0.59	1.42
	+No alcohol		1.26	0.156	0.91	1.76	1.28	0.158	0.91	1.76	1.28	0.166	0.99	1.78
	Labor relations													
		Administrative	1.56	0.067	0.96	2.51	1.55	0.077	0.96	2.51	1.58	0.077	0.92	2.49
		+Teacher	1.06	0.648	0.82	1.37	1.03	0.652	0.82	1.37	1.09	0.647	0.82	1.37
	#Housemates		1.16	0.100	0.97	1.40	1.18	0.100	0.97	1.40	1.16	0.100	0.97	1.40
ZSDS	With (DS)		3.35	<0.001	1.91	5.87	3.33	<0.001	1.90	5.74	3.35	<0.001	1.91	5.87
	Without (DS)		0.92	0.530	0.72	1.18	0.91	0.546	0.72	1.21	0.94	0.530	0.72	1.18
SF-12v2	General health		0.94	<0.001	0.92	0.95	0.93	<0.001	0.92	0.94	0.92	<0.001	0.91	0.94
	Physical functioning		0.97	<0.001	0.95	0.98	0.96	<0.001	0.95	0.97	0.96	<0.001	0.95	0.98
	Physical role		0.98	<0.001	0.97	0.99	0.97	<0.001	0.96	0.98	0.97	<0.001	0.95	0.97
	Emotional role		0.98	0.001	0.97	0.99	0.97	<0.001	0.97	0.99	0.98	<0.001	0.97	0.98
	Bodily pain		0.98	0.011	0.96	0.98	0.98	0.010	0.96	0.99	0.97	0.019	0.96	0.98
	Vitality		0.98	0.014	0.97	0.98	0.99	0.020	0.97	0.99	0.98	0.015	0.97	0.98
	Social functioning		0.99	0.175	0.98	1.00	1.00	0.166	0.98	1.02	0.99	0.175	0.98	1.00
	PCS		0.98	<0.001	0.98	0.99	0.98	<0.001	0.98	0.99	0.99	<0.001	0.98	0.99
	MCS		0.99	0.069	0.99	1.00	1.00	0.072	0.99	1.06	0.99	0.069	0.99	1.00
PSQI	Sleep quality domain		2.84	<0.001	2.01	4.02	2.84	<0.001	2.01	4.02	2.81	<0.001	2.48	4.02
	Sleep latency domain		1.30	0.018	1.04	1.62	1.31	0.017	1.04	1.62	1.32	0.020	1.02	1.62
	Sleep duration domain		1.64	<0.001	1.24	2.15	1.65	<0.001	1.24	2.10	1.67	<0.001	1.27	2.15
	Sleep efficiency domain		5.83	<0.001	3.62	9.40	5.88	<0.001	3.62	9.45	5.89	<0.001	3.65	9.40
	Sleep disturbances domain		5.11	<0.001	3.28	7.95	5.10	<0.001	3.28	7.92	5.13	<0.001	3.31	7.88
	Use of sleeping medication domain		1.56	<0.001	8.58	28.49	1.52	<0.001	8.58	27.89	1.57	<0.001	8.77	28.09
	Daytime dysfunction domain		4.02	<0.001	2.75	5.87	4.08	<0.001	2.78	5.76	4.09	<0.001	2.89	5.89
	Total score for PSQI		1.31	<0.001	1.22	1.41	1.31	<0.001	1.24	1.40	1.35	<0.001	1.22	1.41

The two-sample *t*-test with equal variances was used to compare IPAQ results categorized as active and insufficiently active. BMI (kg/m^2^): body mass index. SF-12v2: 12-item short-form health survey. PCS: physical health component summary. MCS: mental health component summary. PSQI: Pittsburgh Sleep Quality index. IPAQ: The International Physical Activity Questionnaire. * Having one or more underlying medical condition for COVID-19, such as asthma, diabetes, high blood pressure, cancer, a chronic kidney disease, rheumatism, cardiovascular disease, hematological disease or orphan disease. SD: standard deviation. CI: confidence interval. Diff: difference. min: minimum. max: maximum. A significance level of 0.05 was established to evaluate the probability (P) that the mean difference was equal to zero. Mean diff = mean (active)−mean (insufficiently active).

**Table 3:** Simple logistic regression model. Bivariate independent variables associated with the level of physical activity.

Independent Variable		OR	P > z	[95% CI]	
				Min Max	
Sociodemographic	Age	0.99	0.739	0.96	1.02
	Sex male	0.73	0.158	0.47	1.12
	+Sex Female	1.44	0.028	1.04	1.99
	BMI	1.01	0.645	0.96	1.06
	Residence rural area	0.82	0.784	0.20	3.34
	+Urban area	1.21	0.078	0.97	1.51
	Stratification				
	High	1.25	0.456	0.68	2.28
	Medium	0.91	0.733	0.56	1.49
	+Low	1.19	0.249	0.88	1.61
	Marital status				
	Married	1.76	0.018	1.10	2.83
	Separated /widower	1.77	0.083	0.92	3.39
	+Single/Free Union	0.85	0.385	0.61	1.20
	Smoker	0.08	0.021	0.01	0.69
	+No Smoker	1.27	0.027	1.02	1.59
	With * risk	1.36	0.254	0.79	2.34
	+Without * risk	1.13	0.298	0.89	1.44
	Alcohol	0.92	0.708	0.59	1.42
	+No alcohol	1.26	0.156	0.91	1.76
	Labor relations				
	Administrative	1.56	0.067	0.96	2.51
	+Teacher	1.06	0.648	0.82	1.37
	#Housemates	1.16	0.100	0.97	1.40
ZSDS	With (DS)	3.35	<0.001	1.91	5.87
	Without (DS)	0.92	0.530	0.72	1.18
SF-12v2	General health	0.94	<0.001	0.92	0.95
	Physical functioning	0.97	<0.001	0.95	0.98
	Physical role	0.98	<0.001	0.97	0.99
	Emotional role	0.98	0.001	0.97	0.99
	Bodily pain	0.98	0.011	0.96	0.98
	Vitality	0.98	0.014	0.97	0.98
	Social functioning	0.99	0.175	0.98	1.00
	PCS	0.98	<0.001	0.98	0.99
	MCS	0.99	0.069	0.99	1.00
PSQI	Sleep quality domain	2.84	<0.001	2.01	4.02
	Sleep latency domain	1.30	0.018	1.04	1.62
	Sleep duration domain	1.64	<0.001	1.24	2.15
	Sleep efficiency domain	5.83	<0.001	3.62	9.40
	Sleep disturbances domain	5.11	<0.001	3.28	7.95
	Use of sleeping medication domain	1.56	<0.001	8.58	28.49
	Daytime dysfunction domain	4.02	<0.001	2.75	5.87
	Total score for PSQI	1.31	<0.001	1.22	1.41

Data obtained using the simple logistic regression model. BMI (kg/m^2^): body mass index. * Having one or more underlying medical condition for COVID-19, such as asthma, diabetes, high blood pressure, cancer, a chronic kidney disease, rheumatism, cardiovascular disease, hematological disease or orphan disease. SF-12v2: 12-item short-form health survey. PCS: physical health component summary. MCS: mental health component summary. PSQI: Pittsburgh Sleep Quality index. ZSDS: Zung Self-Rating Depression Scale. DS: depressive symptoms. IPAQ: The International Physical Activity Questionnaire. OR: odds ratio. SD: standard deviation. CI: confidence interval. min: minimum. max: maximum. +(cons): reference category.

**Table 4:** Final multiple logistic regression model associated with IPAQ.

Variable		OR	P > z	[95% CI]	
				Min Max	
SF-12v2	General health	0.94	<0.001	0.92	0.97
	Physical functioning	0.98	0.135	0.97	0.98
ZSDS	With (DS)	2.05	0.072	1.93	4.50
PSQI	Sleep efficiency domain	2.41	0.002	1.37	4.22
	Sleep disturbances domain	2.47	0.007	1.28	4.75
	Use of sleeping medication domain	4.34	<0.001	1.99	9.49
	Daytime dysfunction domain	1.80	0.041	1.23	3.19
	Cons	3.22	<0.001	4.78	2.17

Multiple logistic regression model. OR: odds ratio. CI: confidence interval. min: minimum. max: maximum. SF-12v2: 12-item short-form health survey. PSQI: Pittsburgh Sleep Quality index. ZSDS: Zung Self-Rating Depression Scale. DS: depressive symptoms. IPAQ: The International Physical Activity Questionnaire. cons: reference value.

## Data Availability

The data presented in this study are only available on request from the corresponding author. The data are not publicly available because, due to the sensitive nature of the questions asked in this study, participants were assured raw data would remain confidential and would not be shared.
